# Sexual Quality of Life in Gynecological Cancer Survivors in Iran

**DOI:** 10.31557/APJCP.2021.22.7.2171

**Published:** 2021-07

**Authors:** Fariba Yarandi, Ali Montazeri, Elham Shirali, Mona Mohseni, Maliheh Fakehi, Marjan Ghaemi

**Affiliations:** 1 *Department of Gynecology and Obstetrics, Yas Hospital, Tehran University of Medical Sciences, Tehran, Iran. *; 2 *Health Metric Research Center, Iranian Institute for Health Sciences Research, ACECR, Tehran, Iran. *; 3 *Shahid Akbarabadi Hospital, Iran University of Medical Sciences, Tehran, Iran. *; 4 *Vali-e-Asr Reproductive Health Research Center, Tehran University of Medical Sciences, Tehran, Iran. *

**Keywords:** Gynecological cancer-, quality of life, sexual health, sexual quality of life, female (SQOL-F) questionnaire

## Abstract

**Objective:**

Patients with gynecological cancer might suffer from suboptimal sexual quality of life. This cross sectional study aimed to assess the sexual quality of life in the survivors of gynecological cancers and was recruited in a teaching hospital affiliated to Tehran University of Medical Sciences between 2018 and 2020.

**Material and methods:**

The data was collected by a web-based platform with validated self-administered questionnaires including demographic information, and the Sexual Quality of Life-Female (SQOL-F) questionnaire. The data were analyzed using appropriate tests.

**Results:**

Totally, 42% (106) of the participants had a sexual relationship in the last 6 months. The mean (SD) of sexual quality of life score was 46.84 (11.86) with the range of 0-90. The patients with cervical cancer had a worse sexual quality of life in Psychosexual Feelings (P=0.048) and Self-Worthlessness (p=0.036) compared with other gynecological cancers. Sexual quality of life did not improve or worsen over time.

**Conclusion:**

It is concluded poor sexual quality of life need further attention in the traditional societies and healthcare providers are urged to improve their sexual quality of life.

## Introduction

Gynecological cancers including cervical, ovarian, uterine, vaginal and vulvar, represent around 1 in 5 of all cancers diagnosed in women worldwide (world Health Organization, 2018). Cancer in fact is a relationship disease (Brotto, Heiman et al., 2008). It is believed that sexuality and close intimacy have a direct effect on the quality of life of patients with gynecological cancers (Ratner et al., 2010). On the other hand, sexual quality of life may serve as an important part of the general quality of life through and after treatment of gynecological cancers (Juraskova et al., 2013) that could be a major concern in women (Falk and Dizon, 2013).

Sexual dysfunction prevents experiencing satisfaction in sexual activity. It is a multi-factorial and multi-dimensional issue combining biological, psychological and interpersonal determinants (Laumann et al., 1999) that recognized for reducing sexual desire, difficulties in arousal and maintaining desire, orgasm dysfunction, and sexual pain (Basson et al., 2010). Treatment modalities in cancers such as surgery, chemoradiotherapy, and/or changes in the sex hormone levels, may lead to sexual dysfunction and because of direct effect on the female sexual organs (Goncalves, 2010) it effects many aspects such as sexual arousal and inadequate lubrication-swelling response of sexual excitement (Brotto et al., 2008; Association, 2018). Pain during sex or dyspareunia is the other major sexual dysfunction that may cause by vaginal dryness due to the lack of vaginal estrogen (Ratner et al., 2010). 

The sexual quality of life of women with gynecological cancers is moderate and needs support especially from family or friends (Golbasi and Erenel, 2012). Despite sufficient information about the prevalence of sexual dysfunction among gynecological cancer survivors, the progress to find the right solution has lagged. It may be due to a lack of data about the importance of sexuality because few studies have evaluated the potential implications of sexual dysfunction in these women (Levin et al., 2010). 

Fortunately, recent adventures on electronic communications allow easier data collection via online platforms that could simplify this process and accelerate information transfer between the patients and the health care providers (Richter et al., 2008). Although, using electronic questionnaires are becoming popular among investigators, there are few studies about quality of life with data collection among electronic devices (Richter et al., 2008; Aktas et al., 2015). Also, in traditional societies, where talking about sex life is with shame and even unacceptable receiving information via online questionnaire may be a good alternative.

Accordingly, this study was designed to determine the sexual quality of life in the survivors of gynecological cancers using a new mobile device technology, because it seems that the prevention and treatment of sexual dysfunction may have important secondary benefits for cancer survivors (Levin et al., 2010).

## Materials and Methods

*Design and patients*

This was a cross-sectional study to assess the sexual quality of life using an online questionnaire. The study included a sample of women survivors with a confirmed history of gynecological cancers referred to a teaching hospital affiliated to Tehran University of Medical Sciences, Tehran, Iran between 2018 and 2020. The participant’s initial data were obtained from the electronic health records of the hospital. 

*Eligibility criteria included the following conditions*

Having a sexual partner, at least six months from completion of treatment, no recurrence of the disease, ability to understand and communicate in the current language of the region, and having adequate electronic literacy. Participants who had no sex during the last 6 months or with a history or current psychiatric and severe medical conditions and also using the medications that might have impacted sexual functioning, such as antidepressants and anxiolytics in the past 6 months, were excluded. Indeed the history of hormone replace therapy by any mode of conception were excluded either.

*Ethical consideration*

This study was approved by the institutional Ethical Committee (IR.TUMS.MEDICINE.REC.1397.214) affiliated to Tehran University of Medical Sciences. Informed consent was taken from all participants via online form. This trial was conducted according to the principles of the Helsinki Declaration. 

*Data collection*

An online questionnaire was designed to collect the data. A professional team was designed the platform with multiple layers of security to make sure that the data remains private and secure. A qualified nurse made a telephone call to each participant to introduce the study. After initial verbal consent to participate in the study, a direct link to the questionnaire was sent via Short Message Service to their cell phone. 

The computer program was designed to allow only one response from any unique phone number and computer IP. Therefore, the patients could complete the forms only one time. As we called any single patients before completing the questionnaire, we are almost sure these patients themselves, completed the forms.

By clicking on the link, they were directed to a web site. In the beginning, a single question was asked: “Do you have had sexual activity in the last 6 months?” By selecting “yes” they would be redirected to the main page including a consent form (by clicking a box the cases accepted to participate in the study), the demographics and the Sexual Quality of Life-Female (SQOL-F) questionnaire. 

The questions were answered by ticks for the appropriate answer. The system notified the participant if a question was missed. Assistance could be provided by family members. Once completed they encouraged to save their responses by click on the finish bottom. The data were properly secured when stored on a computer and a password accessed server. Data was collected as a spreadsheet and remained anonymous with no information linking questionnaires to the participants. 

*Questionnaires*

1. Demographic information: This included items on age, education, occupation, and income, time since diagnosis and information about the type of cancer, treatment type and the date of their last medical, surgical or hormonal management.

2. The Sexual Quality of Life-Female (SQOL-F) questionnaire is specifically assessed the relationship between female sexual dysfunction and quality of life that was developed by Symonds et al., (2005). It consists of 18 parts and each item is rated on a six-point response (completely agree to completely disagree). The response categories could be scored 0 to 5 giving a total score of 0–90 (Symonds et al., 2005). Higher score indicates better quality of life. The basis for the generation of this questionnaire was Spitzer’s Quality of Life (QOL) model that involved physical, emotional, psychological and social components. The Iranian version of this questionnaire was validated by Maasoumi et al. with good psychometric properties (Maasoumi et al., 2013) with Cronbach’s alpha coefficient 0.73 and intraclass correlation coefficient 0.88. This article was the first and one of the most validated translated version of SQOL-F in Iran. The descriptive statistic for the SQOL-F subscales in this study was compared with our results.

*Data analysis*

Descriptive analysis was used to describe the data. Qualitative variables were presented as number and percentage, and quantitative variables as mean (SD). Continuous variables were compared using the one-way analysis of variance (ANOVA) with post hoc analysis, and categorical variables were examined using Pearson’s chi-square test with continuity correction. P value <0.05 was considered statistically significant. Data were analyzed using SPSS 24 (SPSS Inc, Chicago, Illinois).

## Results

In this study, 106 women were recruited and completed the questionnaires successfully. Among the participants, 39.6%, 36.7% and 23.5% had uterine, ovarian and cervical cancer, respectively. None of the participants experienced vulvar or vaginal cancers. The mean (SD) age was 52.9 (11.8) ranging from 26 to 75 years. 

Surgery, chemotherapy, and radiotherapy were used as the treatment modalities in 71 (66.9%), 49 (46.2%), and 35 (33.0%) cases, respectively. The basic characteristics of the patients are presented in Table 1.

The SQOL-F subscales scores in this study and the reference study (Maasoumi et al., 2013) in healthy females in Iran are shown in Table 2. The sexual quality of life is below 50% in most aspects. The patients with cervical cancer had a worse sexual quality of life in psychosexual feelings (P=0.048) and self-worthlessness (p=0.036) compared with other cancers. The results of the SQOL-F questionnaire by cancer type are listed in Table 3. 

Table 4 demonstrates the SQOL-F subscales by cancer treatment. As shown, there is not any significant differences between the sexual quality of life scores and the different treatment modalities.

In evaluation performed to consider the relationship between the date of diagnosis and the total score of sexual quality of life, no significant difference (p=0.193) was observed.

**Table 1:** Basic Demographics and Treatment Characteristics of the Patients

	Total (n=106)	Uterine (n=42)	Ovary (n=39)	Cervix (n=25)	P*
Age (years)					
Mean (SD)	52.9 (11.8)	56.7 (9.2)	47.9 (11.5)	54.2 (13.7)	0.002
Education (%)					0.36
Illiterate	10.3	11.9	7.6	12	
Primary	18.8	28.5	12.8	12	
Secondary	51-8.5	42.8	61.5	52	
Higher	18.8	16.6	17.9	24	
Working Status (%)					0.117
Working	14.2	7.1	17.9	20	
Housewife	85.8	92.6	82.1	80	
Income (%)					0.309
Poor/fair	87.7	90.9	87.2	84	
Good	12.3	9.5	12.8	16	
Time since diagnosis (years) (%)					0.255
<1 year	17.4	21.3	18.8	9.1	
Between 1 to 5 years	78.4	73.4	76.6	89.1	
> 5 years	4.2	5.3	4.7	1.8	
Treatment*					< 0.001
Surgery	44.3	40.5	64.1	20	
Chemoradiotherapy	23.6	26.2	2.6	52	
Surgery & Chemotherapy	15.1	7.1	33.5	0	
Surgery & Chemoradiotherapy	13.2	16.7	0	28	
Surgery & Radiotherapy	9.5	0	0	3.8	

*, P-values derived from one-way analysis of variance for continuous variables, and Pearson's chi-square test for categorical variables; **, Derived from analysis of covariance (age as covariate).

**Table 2:** Sexual Quality of Life Scores as Measured by the SQOL-F in Ccancer and Non-Cancer Patients*

	Cancer		Non-cancer**	
	Range	Mean±SD*	Range	Mean±SD
Psychosexual feelings	7-35	15.90±8.3	7-42	28.2±7. 9
Sexual and relationship satisfaction	5-25	17.12±5.6	5-30	24.3±5.4
Self-worthlessness	3-15	6.48±3.4	3-18	15.4±3.7
Sexual repression	3-15	8.17±2.6	3-18	13.9±4.1
Total score	23-82	46.84±1.8	18-108	86.4±1.7

*, Higher values indicate better conditions; **, Derived from the study by

**Table 3:** Results of SQOL-F Questionnaire by Cancer Diagnosis

	Uterine* (n=42)	Ovary (n=39)	Cervix (n=25)	P
	Mean±SD	Mean±SD	Mean±SD	
Psychosexual Feelings	15.9±8.3	16.3±8.7	18.8±9.3	0.048
Sexual and Relationship Satisfaction	17.1±5.6	17.3±6	15±5.9	0.075
Self-Worthlessness	6.4±3.4	6.8±3.4	7.6±3.9	0.036
Sexual Repression	8.1±2.6	8.4±2.6	8.2±2.2	0.638
Total score	46.8±11.8	48.2±11.9	49.7±13.2	0.097

*, Higher values indicate better conditions

**Table 4:** Results of SQOL-F Questionnaire by Cancer Treatment

	Surgery* (n=71)	Chemotherapy (n=49)	Radiotherapy (n=35)	P
	Mean±SD	Mean±SD	Mean±SD	
Psychosexual Feelings	15.0±7.9	16.8±8.9	16.5±8.9	0.723
Sexual and Relationship Satisfaction	17.8±5.6	17.1±5.1	16.7±5.4	0.846
Self-Worthlessness	6.1±3.2	7.0±3.9	7.0 ±3.8	0.665
Sexual Repression	8.0±2.5	8.6±2.8	8.3±2.3	0.644
Total score	46.2±11.1	49.1±13.0	48.0±12.1	0.489

*Higher score indicates better conditions

## Discussion

Sexual dysfunction caused by gynecological cancers are not often discussed or expressed within relationships (Juraskova et al., 2003) and is far from the expert’s attention. This is especially true in traditional and religious area like Middle East that the women are still not comfortable talking about their sexual life. In addition, the clinicians may feel uncomfortable discussing sexual health issues due to the lack of training and embarrassment (Abdolrasulnia et al., 2010). 

The gynecological cancer survivors encounter altered body Image and sexuality after treatment (Sacerdoti et al., 2010). As in this study, the participants were found to have a poorer sexual quality of life on all scales compare with the general population and also sex related distress experienced by survivors was higher than non-affected women. Psychosexual feelings and self-worthlessness were the significant subscales in sexual issues that were lower by cancer diagnosis in our study. In a search in PubMed, only two suitable articles were found as a proper reference of normal female score of SQOL-F in Iran (Maasoumi et al., 2013, Pakpour et al., 2013). We used the first article as a reference. Because it was more compatible in scoring method of the subgroups with our study.

This fact confirms that such studies are very rare in Iran and that talking about sexual issues is still considered as taboo.

Andersen and van Der Does (1994) reported a higher frequency of sexual dysfunctions in patients with gynecological cancer in comparison with healthy women. This may be due to the chemoradiotherapy that decreases libido and negatively affects orgasm and also surgical treatment may lead to loss of sexual desire.

Women with vulvar cancers are at high risk of sexual dysfunction compared with healthy controls (Aerts et al., 2014) but in our study, none of the participants with vulvar or vaginal cancer had sexual relationship which could be due to the lower percentage and quitting sexual relationships in this population.

Cervical cancer is the most common gynecological cancer. As mentioned previously, the cases with cervical cancer in our study due to the late attendance and various treatment modalities including chemoradiotherapy had a worse sexual quality of life compared with other gynecological cancers. In this context, appropriate screening methods such as vaccine and pap-smear are recommended to prevent cervical cancer. 

Compared with healthy women, ovarian cancer survivors experience less libido, vaginal dryness and dyspareunia (Liavaag et al., 2008). The resultant menopausal symptoms due to the removal of the ovaries can result in diminished sexual desire (Hughes et al., 1991). The women with endometrial cancers are older than the other participants even not significantly. They also experience more sexual difficulties when compared with healthy women (Aerts et al., 2015). These results is in line with our cases.

To improve the sexual quality of life, it is important to identify which patients may be suffering from sexual health issues at regular intervals that make the online questionnaires an optimal option. Online questionnaires may give a comprehensive picture of the population’s health status. It provides opportunities to enhance patient care and facilitate clinical research (Cohen, 1999). This method is also acceptable in studies related to health quality of life in cancers. Nearly most of our expected participants filled the questionnaire after the first call or one reminder call. 

Online sampling also allowed us to access hard to reach population, although, clinical information about this way is limited. Future studies should consider assessing supervised interventions in settings other than home-based. It was difficult for many of our patients who lived all over the country to have a face to face visit, and this method improved access due to its ease and prevention of travel and spending more time and money.

In traditional societies, most women do not desire to talk about sex especially those who are in middle age. On the other hand, cancer treatments have a profound effect on them, which in addition to possible premature menopause, makes them feel ashamed. Most of these women blame themselves for losing fertility based on the belief that women should be productive. They prefer not to talk face to face with the doctor. In this regard, online questionnaires can remove the embarrassment in them and help them to talk with their health care providers more easily.

We used a validated instrument to assess the sexual quality of life. Most studies in this region are focused on breast cancer. But studies about quality of life in gynecological cancer are scarce. Indeed, we successfully used an online method to collect data that could help to reevaluate the patients for long term and interventional studies.

The main limitation of our study was the low sample size. Indeed, we did not have an age-matched control group. Although designing the prospective cohort studies and long term follow up are recommended.

In conclusion, women with gynecological cancers who have decreased sexual quality of life needing further attention from clinicians. It is important to note that we could find a significant difference between the gynecological cancers in the score of the psychosexual feelings and self-worthlessness that were lower in cervical cancer. 

## Author Contribution Statement

A.M, Project development; F.Y, Project management; M.G, Manuscript writing; E.S, Data collection and management; M.M, Manuscript editing, data management; M.F, Manuscript editing; All authors approved the submitted version (and any substantially modified version that involves the author’s contribution to the study.

## Availability of data (if apply to your research)

Data will be available upon request.
